# Association Between Nutrition Literacy and Overweight/Obesity of Adolescents: A Cross–Sectional Study in Chongqing, China

**DOI:** 10.3389/fnut.2022.893267

**Published:** 2022-05-12

**Authors:** Shengping Li, Yuzhao Zhu, Mao Zeng, Zhourong Li, Huan Zeng, Zumin Shi, Yong Zhao

**Affiliations:** ^1^Department of Nutrition and Food Hygiene, School of Public Health and Management, Chongqing Medical University, Chongqing, China; ^2^Research Center for Medicine and Social Development, Department of Nutrition and Food Hygiene, School of Public Health and Management, Chongqing Medical University, Chongqing, China; ^3^Department of Communicable Disease Control and Prevention, Chengdu Shuangliu District Disease Prevention and Control Center, Sichuan, China; ^4^Chongqing Key Laboratory of Child Nutrition and Health, Children's Hospital of Chongqing Medical University, Chongqing, China; ^5^Human Nutrition Department, College of Health Sciences, QU Health, Qatar University, Doha, Qatar

**Keywords:** nutrition literacy, overweight, obesity, body mass index, adolescents, Chongqing

## Abstract

**Objective:**

The burden of overweight and obesity in adolescents is increasing rapidly. This study aimed to assess the association between nutrition literacy and overweight/obesity among adolescents in China.

**Methods:**

This cross–sectional online study involving adolescents aged 10–18 years was conducted in September 2020 in 239 schools in Chongqing China. Overweight and obese adolescents were determined based on the International Obesity Task Force's recommended age–sex specific body mass index cutoff points. Nutrition literacy was measured using the “Nutrition literacy scale for middle school students in Chongqing (CM–NLS).” The CM–NLS included three subdomains (functional nutritional literacy, interactive nutrition literacy, and critical nutrition literacy). Multinomial logistic regression model was used to examine the association.

**Results:**

A total of 18,176 adolescents (49.8% girls) were included. The prevalence of overweight and obesity was 9.6% and 17.0%, respectively. Compared with those having a low nutrition literacy score (below median), those with a high score were less likely to be overweight and obese. The odds ratio (95% CI) for overweight was 0.87 (0.79–0.97) (nutrition literacy) and 0.81 (0.73–0.90) (functional nutritional literacy). The corresponding figures for obesity were 0.84 (0.77–0.91) and 0.73 (0.67–0.80), respectively. Significant interaction existed between grade and nutrition literacy. The inverse association between nutrition literacy and overweight/obesity was significant among those in senior school but not among those in junior high school.

**Conclusion:**

Nutrition literacy was inversely associated with overweight/obesity among adolescents, especially those attending senior high schools.

## Introduction

Overweight and obesity are some of the most serious public health problems today. The World Health Organization declared that over 340 million children and adolescents were overweight or obese worldwide in 2016. The number of obese children and adolescents (aged 5–19 years) had risen ten–fold over the past four decades (1), and the rising trends is accelerating in parts of Asia (2). According to the Report on Nutrition and Chronic Diseases in China (2020), the prevalence of overweight/obesity among children and adolescents was nearly 20% (3). Being overweight or obese during childhood and adolescence is associated with adverse health consequences, such as being more likely to be obese in adulthood (4), and is a major risk factor for chronic diseases (5), including diabetes, cardiovascular disease, and some cancers (6, 7).

Overweight and obesity can be prevented by choosing healthier foods and regular physical activity. Adolescence is a crucial stage for developing dietary habits, influenced by nutrition knowledge and other factors (8). Nutrition literacy (NL) is associated with overall food habits (9, 10). It is defined as obtaining, understanding, and using correct nutrition information and nutrition knowledge to make healthy food choices (11). Krause et al. (12) classified NL into three subdomains, namely, functional NL (FNL), the ability to obtain and process nutrition information to improve decisions about nutrition; interactive NL (INL), the ability to utilize different forms of communication to obtain and apply relevant nutrition information; and critical NL (CNL), the ability to critically assess and reflect on nutrition information.

Nutrition literacy is a new field of study, and its concept originates from health literacy (13). Existing studies have shown the correlation of children's health literacy with overweight and obesity. A recent review based on 32 studies conducted in children (*n* = 4) and adults (*n* = 28) found that health literacy is a determinant in obesity control (14). A study of 162,209 sixth–grade (11–12 years old) students in Taiwan showed that students with higher health literacy are less likely to be obese (15). A study from Australia suggested that interventions on adolescent obesity should improve their NL and skills (16).

Existing studies on the association between NL and overweight/obesity were primarily conducted in adults (17). One study has revealed no association between NL with BMI (18). However, another research has shown that lower NL is more problematic for weight loss (19). Few studies have explored the relationship between NL and overweight/obesity. An enhanced understanding of the effect of adolescents' NL on overweight/obesity may play a positive role in obesity prevention and control. One of our previous studies assessed the determinants of NL and found that ethnicity, grade, residence, whether receiving school meal support from the government, primary caregiver, parents' education level, and BMI are related NL among adolescents [20]. The current study aimed to explore the relationship between NL and overweight/obesity among adolescents in Chongqing China. And we hypothesized that the low levels of NL and three subdomains were all associated with a high prevalence of overweight/obesity among adolescents.

## Materials and Methods

### Study Design and Sample

This cross–sectional online study was conducted in September 2020. We selected 29 of 38 administrative areas and 239 schools in Chongqing as the survey sites. The convenience sampling method was utilized with the online survey platform “Questionnaire Star,” which is a professional online survey platform in China. Then, the questionnaire link or QR code was sent to each regional school health workgroup through the Chongqing Municipal Education Commission. The school health worker forwarded the questionnaire to the class teacher of grades 7, 8, 10, and 11, and the teacher guided the students to complete the questionnaire. Students completed the questionnaire anonymously and independently in 10–15 min.

A total of 21,084 students (grades 7, 8, 10, and 11) participated in the survey. We excluded participants with extreme values of NL score (NL score < 5% centile, or NL score > 95% centile, *n* = 917), extreme BMI values (BMI < 1% centile, or > 99% centile, *n* = 484), and those reported “don't know” of their parents' education (*n* = 1,507). Finally, 18,176 participants aged 10–18 years were included in the study. The study was approved by the Ethics Committee of Chongqing Medical University (approval number: 2021041). All participants were informed about the study, and consents were obtained before the survey.

### Outcome Variable: Overweight and Obesity

Height and weight were self–reported by the students. According to the regulations in China (20), schools need to organize physical examinations for students at the start of the school year in the spring and autumn. Thus, students' self–reported height and weight are more likely to be valid unless intentional over– or under–reporting of weight. Body mass index (BMI) was calculated and classified as underweight, normal, overweight, and obese based on the International Obesity Task Force cutoffs (21).

### Exposure Variable: NL Score

Nutrition literacy was measured based on the “Nutrition literacy scale for middle school students in Chongqing (CM–NLS).” The development of the scale is described elsewhere (22). It includes three subdomains (FNL, INL, and CNL). The scale had been tested for its validity (KMO = 0.916) and reliability (Cronbach's α of the total scale and three subdomains = 0.849, 0.826, 0.942, 0.938, respectively) among 462 middle school students (23). The validity (KMO = 0.945) and reliability (Cronbach's α of the total scale and three subdomains = 0.899, 0.792, 0.925, 0.927, respectively) test of the 18,176 adolescents was also conducted.

The CM–NLS comprises 52 items (the specific items of FNL/INL/CNL are shown in the Supplementary Material). FNL includes 35 items with three skills, namely, obtain skill, understand skill, and apply skill. INL includes 5 items with interact skill. CNL comprises 12 items with media literacy and critical skill. To make the score comparable, we converted it into a centesimal for a sum score (0 to 100 points) of the total and three subdomains, with a higher score indicating a better NL level. The scores of NL, FNL, INL, and CNL were divided into low and high levels based on their median scores (61.9, 68.7, 70.0, and 45.8, respectively).

### Covariates

The following variables were treated as covariates: gender, grade (junior high school included grade 7 and 8, whereas senior high school included grade 10 and 11), ethnicity (Han and minority), residence (urban and rural), primary caregiver (parents and others), and parent's education (low, elementary school, and below; medium, junior high school; and high, high school, or above). As a high proportion of students lived in school (boarding school), we treated it as a covariate.

### Statistical Analysis

Descriptive statistics included frequencies and percentages of research variables. The Chi–square test was used to analyze the associations between the category of BMI and NL, as well as other covariates. Multinomial logistic regression model was used to analyze the association between NL and BMI categories. Two models were established as follows: model 1 was not adjusted; and model 2 was adjusted for gender, grade, ethnicity, boarding school, residence, primary caregiver, and parent's education. We tested multiplicative interaction between NL and demographic characteristics (gender, grade, ethnicity, boarding in school, residence, and primary caregiver) by adding the product of the variables in a multivariable model.

All analyses were performed using STATA version 16.0 (STATA Corporation, College Station, TX, USA). Statistical significance was considered when *p* < 0.05 (two–sided).

## Results

### Sample Description

Table 1 summarizes the demographic characteristics of 18,176 middle school students. The median age of students was 14 years (range 10–18 years). In the sample, 50.2% of the participants were boys, 43.2% were junior high school students, 88.8% were of Han ethnicity, 65.6% were in boarding school, 48.6% lived in urban area, and 71.3% of the participant's primary caregivers were parents. The prevalence of overweight and obesity was 9.6% and 17.0%, respectively. Across the levels of NL, the participants of Han nationality who were living in an urban area had a higher rate of of NL, FNL, INL, and CNL. Girls and participants whose primary caregivers were parents also had a higher rate of elevated levels of NL and FNL. Compared with senior high school students, junior high school students had a higher level of all domains of NL. Students who were in boarding school had higher NL levels than their counterparts. And the results showed that the levels of NL varied between different levels of parents' education (*p* < 0.001).

**Table 1:** Distribution by levels of nutrition literacy across demographic characteristics.

**Factor**	**Total** ***N* = 18,176**	**Nutrition Literacy (NL)**		***p* ^a^**	**Functional Nutrition Literacy (FNL)**		***p* ^a^**	**Interactive Nutritional Literacy (INL)**		***p* ^a^**	**Critical Nutrition Literacy (CNL)**		***p* ^a^**
		**Low** **N = 9,434**	**High** ***n* = 8,742**		**Low** ***n* = 9,753**	**High** ***n* = 8,423**		**Low** ***n* = 9,134**	**High** ***n* = 9,042**		**Low** ***n* = 8,603**	**High** ***n* = 9,573**	
**Gender**													
Boy	9118 (50.2)	4802 (50.9)	4316 (49.4)	0.040	5047 (51.7)	4071 (48.3)	<0.001	4623 (50.6)	4495 (49.7)	0.220	4300 (50.0)	4818 (50.3)	0.640
Girl	9058 (49.8)	4632 (49.1)	4426 (50.6)		4706 (48.3)	4352 (51.7)		4511 (49.4)	4547 (50.3)		4303 (50.0)	4755 (49.7)	
**Grade**													
Junior high school	7858 (43.2)	4456 (47.2)	5862 (67.1)	<0.001	4378 (44.9)	5940 (70.5)	<0.001	4424 (48.4)	5894 (65.2)	<0.001	4818 (56.0)	5500 (57.5)	0.049
Senior high school	10318 (56.8)	4978 (52.8)	2880 (32.9)		5375 (55.1)	2483 (29.5)		4710 (51.6)	3148 (34.8)		3785 (44.0)	4073 (42.5)	
**Ethnicity**													
Han	16137 (88.8)	8044 (85.3)	8093 (92.6)	<0.001	8263 (84.7)	7874 (93.5)	<0.001	7876 (86.2)	8261 (91.4)	<0.001	7570 (88.0)	8567 (89.5)	0.001
Minority	2039 (11.2)	1390 (14.7)	649 (7.4)		1490 (15.3)	549 (6.5)		1258 (13.8)	781 (8.6)		1033 (12.0)	1006 (10.5)	
**Boarding school**													
No	6251 (34.4)	2696 (28.6)	3555 (40.7)	<0.001	2615 (26.8)	3636 (43.2)	<0.001	2823 (30.9)	3428 (37.9)	<0.001	2909 (33.8)	3342 (34.9)	0.12
Yes	11925 (65.6)	6738 (71.4)	5187 (59.3)		7138 (73.2)	4787 (56.8)		6311 (69.1)	5614 (62.1)		5694 (66.2)	6231 (65.1)	
**Residence**													
Urban	8839 (48.6)	4111 (43.6)	4728 (54.1)	<0.001	4156 (42.6)	4683 (55.6)	<0.001	4362 (47.8)	4477 (49.5)	0.018	4000 (46.5)	4839 (50.5)	<0.001
Rural	9337 (51.4)	5323 (56.4)	4014 (45.9)		5597 (57.4)	3740 (44.4)		4772 (52.2)	4565 (50.5)		4603 (53.5)	4734 (49.5)	
**Primary caregiver**													
Parents	12961 (71.3)	6579 (69.7)	6382 (73.0)	<0.001	6846 (70.2)	6115 (72.6)	<0.001	6456 (70.7)	6505 (71.9)	0.060	6072 (70.6)	6889 (72.0)	0.040
Others^b^	5215 (28.7)	2855 (30.3)	2360 (27.0)		2907 (29.8)	2308 (27.4)		2678 (29.3)	2537 (28.1)		2531 (29.4)	2684 (28.0)	
**Father's education**													
Low^c^	4055 (22.3)	2446 (25.9)	1609 (18.4)	<0.001	2571 (26.4)	1484 (17.6)	<0.001	2232 (24.4)	1823 (20.2)	<0.001	2042 (23.7)	2013 (21.0)	<0.001
Medium^d^	9284 (51.1)	4837 (51.3)	4447 (50.9)		5036 (51.6)	4248 (50.4)		4493 (49.2)	4791 (53.0)		4389 (51.0)	4895 (51.1)	
High^e^	4837 (26.6)	2151 (22.8)	2686 (30.7)		2146 (22.0)	2691 (31.9)		2409 (26.4)	2428 (26.9)		2172 (25.2)	2665 (27.8)	
**Mother's education**													
Low^c^	5711 (31.4)	3475 (36.8)	2236 (25.6)	<0.001	3655 (37.5)	2056 (24.4)	<0.001	3134 (34.3)	2577 (28.5)	<0.001	2862 (33.3)	2849 (29.8)	<0.001
Medium^d^	8313 (45.7)	4172 (44.2)	4141 (47.4)		4350 (44.6)	3963 (47.0)		3989 (43.7)	4324 (47.8)		3888 (45.2)	4425 (46.2)	
High^e^	4152 (22.8)	1787 (18.9)	2365 (27.1)		1748 (17.9)	2404 (28.5)		2011 (22.0)	2141 (23.7)		1853 (21.5)	2299 (24.0)	

a *Chi–square test showing distribution by levels of NL across demographic characteristics*.

b *Grandparents and relatives*.

c *Elementary school and below*.

d *Junior high school*.

e *High/technical/vocational/college/undergraduate and above*.

### Association Between NL and BMI Categories

**Figure 1:**
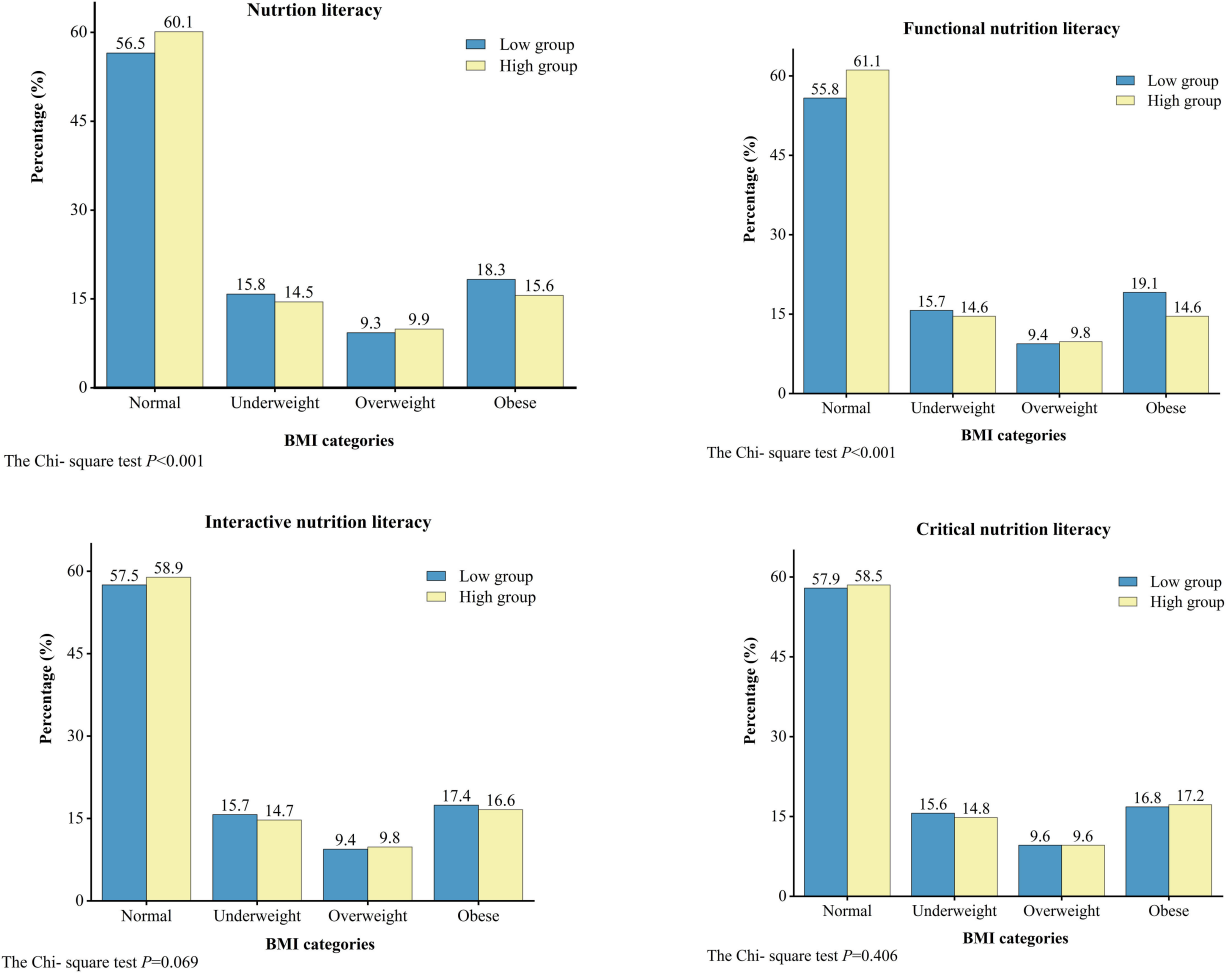
Distribution of underweight, overweight, and obese in different nutrition literacy levels.

The results of Chi–square test for two–by–two comparisons showed that participants with high levels of NL and FNL had a lower prevalence of obesity than those with low levels (Figure 1). The prevalence of obesity was higher in low NL (18.3%) and low FNL (19.1%) groups than in high NL (15.6%) and high FNL (14.6%) groups, respectively (*p* < 0.001). However, no difference existed in the prevalence of obesity by levels of INL and CNL.

In the fully adjusted model, compared with those having lower NL, those with higher NL were less likely to be underweight or overweight/obese (Table 2). The odds ratios (95% CI) for underweight, overweight, and obese were 0.91 (95% CI = 0.83–0.99), 0.87 (95% CI = 0.79–0.97), and 0.84 (95% CI = 0.77–0.91). Higher FNL was inversely associated with overweight (0.81; 95% CI 0.73–0.90) and obesity (0.73; 95% CI = 0.67–0.80). However, INL and CNL were not associated with underweight or overweight/obesity.

**Table 2:** Multinomial logistic regression model of the association between BMI categories and nutrition literacy.

**Factor**	**Model 1** ^**a**^		**Model 2** ^**b**^	
	**Odds Ratio (95% CI)**	***p*–value**	**Odds Ratio (95% CI)**	***p*–value**
**Nutrition Literacy (NL)**				
Normal (Ref)				
Underweight	0.86 (0.79–0.93)	<0.001	0.91 (0.83–0.99)	0.029
Overweight	1.00 (0.90–1.10)	0.954	0.87 (0.79–0.97)	0.012
Obese	0.80 (0.74–0.86)	<0.001	0.84 (0.77–0.91)	<0.001
**Functional Nutrition Literacy (FNL)**				
Normal (Ref)				
Underweight	0.85 (0.78–0.93)	<0.001	0.92 (0.84–1.00)	0.060
Overweight	0.96 (0.86–1.06)	0.400	0.81 (0.73–0.90)	<0.001
Obese	0.70 (0.64–0.76)	<0.001	0.73 (0.67–0.80)	<0.001
**Interactive Nutritional Literacy (INL)**				
Normal (Ref)				
Underweight	0.91 (0.84–0.99)	0.035	0.95 (0.87–1.04)	0.252
Overweight	1.01 (0.91–1.12)	0.831	0.95 (0.86–1.06)	0.365
Obese	0.93 (0.86–1.00)	0.065	0.94 (0.87–1.02)	0.148
**Critical Nutrition Literacy (CNL)**				
Normal (Ref)				
Underweight	0.93 (0.86–1.01)	0.107	0.94 (0.87–1.03)	0.171
Overweight	0.99 (0.89–1.09)	0.764	0.96 (0.86–1.06)	0.410
Obese	1.01 (0.93–1.09)	0.855	1.02 (0.95–1.12)	0.495

a *Model 1 unadjusted*.

b *Model 2 adjusted for gender, grade, ethnicity, primary caregiver, parent's education, boarding school, and residence*.

### Subgroup Analyses of the Association Between NL and BMI Categories

No interactions of NL, FNL, INL, and CNL with gender, ethnicity, boarding in school, residence, and primary caregiver in relation to overweight/obesity were observed (Table 3). A significant NL (*p* = 0.009), FNL (*p* = 0.003), and grade interaction was observed. In participants from senior high school, NL and FNL were inversely associated with overweight/obesity. However, no such association was found in junior high school students.

**Table 3:** Subgroup analyses of the association between BMI categories and nutrition literacy.

**Factor**	**NL**	***p* ^a^**	**FNL**	***p* ^a^**	**INL**	***p* ^a^**	**CNL**	***p* ^a^**
	**High vs. Low**		**High vs. Low**		**High vs. Low**		**High vs. Low**	
**Gender**								
Boy	0.85 (0.78–0.92)^**^	0.333	0.82 (0.75–0.90) ^**^	0.857	0.94 (0.87–1.03)	0.639	0.95 (0.87–1.03)	0.332
Girl	0.90 (0.83–0.98)^*^		0.81 (0.74–0.88) ^**^		0.95 (0.88–1.04)		1.02 (0.93–1.11)	
**Grade**								
Junior high school	0.93 (0.86–1.01)	0.009	0.83 (0.76–0.90) ^**^	0.379	1.02 (0.94–1.10)	0.003	1.02 (0.94–1.10)	0.163
Senior high school	0.78 (0.71–0.86)^**^		0.77 (0.70–0.85) ^**^		0.85 (0.77–0.93) ^**^		0.93 (0.85–1.02)	
**Ethnicity**								
Han	0.87 (0.82–0.93)^**^	0.937	0.80 (0.75–0.85) ^**^	0.436	0.93 (0.87–0.99) ^*^	0.198	0.98 (0.92–1.04)	0.652
Minority	0.87 (0.71–1.05)		0.88 (0.71–1.08)		1.05 (0.88–1.27)		1.02 (0.85–1.21)	
**Boarding school**								
No	0.87 (0.79–0.97) ^*^	0.958	0.80 (0.72–0.88) ^**^	0.660	1.03 (0.93–1.14)	0.061	0.95 (0.86–1.05)	0.393
Yes	0.87 (0.81–0.94)		0.81 (0.75–0.88) ^**^		0.90 (0.84–0.97) ^**^		1.00 (0.93–1.08)	
**Residence**								
Urban	0.87 (0.80–0.95) ^*^	0.989	0.82 (0.75–0.90) ^**^	0.706	0.95 (0.87–1.04)	0.945	0.97 (0.89–1.06)	0.801
Rural	0.87 (0.79–0.94) ^**^		0.79 (0.73–0.87) ^**^		0.94 (0.86–1.02)		0.99 (0.91–1.07)	
**Primary caregiver**								
Parents	0.85 (0.79–0.91) ^**^	0.349	0.79 (0.74–0.85) ^**^	0.663	0.91 (0.85–0.98) ^**^	0.098	1.01 (0.90–1.04)	0.486
Others	0.92 (0.82–1.02)		0.83 (0.74–0.93) ^*^		1.04 (0.93–1.16)		0.97 (0.90–1.13)	

*Model adjusted for gender, grade, ethnicity, primary caregiver, parent's education, boarding school, and residence*.

a *p for interaction*.

* *p < 0.05*,

** *p < 0.01*.

An association between NL and overweight/obesity was found across genders, residence (urban and rural), and participants from the Han nationality, not boarding in school, and primary caregiver was parents. For the three subdomains, the association between FNL and overweight/obese was similar across genders, grade, ethnicity, boarding school, residence, and primary caregiver. An association between INL and overweight/obesity was found among senior high school students, Han ethnicity, in boarding school, and the primary caregiver was parents. However, no significant association between CNL and overweight/obesity existed across genders, grade, ethnicity, boarding in school, residence, and primary caregiver.

## Discussion

Given the importance of promoting NL among adolescents and in light of surging obesity levels, this cross–sectional study in a large population–based sample examined the relative contributions of NL to overweight/obesity among middle school students in Chongqing. Our results showed that the prevalence of overweight and obesity in low (27.6%) and high (25.5%) NL groups was higher than the national average (19.0%) based on the Report on Nutrition and Chronic Diseases in China (2020) (3). Moreover, consistent with our hypothesis, NL and FNL was inversely associated with the prevalence of overweight/obesity. But no difference was observed in the prevalence of underweight or overweight/obesity by levels of INL and CNL. These findings, along with several other interesting results, raise theoretical references for interventions of preventing and controlling obesity of adolescents.

To the best of our knowledge, few studies have examined the association between NL of adolescents and BMI or overweight/obesity. Our previous studies found that BMI is a determinant of NL among middle school students in Chongqing, China (24). Using multinomial logistic regression model and subgroup analyses, our findings suggested inverse relationships between NL, FNL, and overweight/obesity, different from some previous studies (11, 18). Several explanations were considered for these findings. Previous researchers have demonstrated that NL significantly affects healthy eating behavior (25, 26) and positively changes the food habits (9) and choices (27) of the adolescents. Overall diet quality was shown to decrease with age as adolescents (28), but it can be mitigated with the attainment of NL (29, 30). Additionally, NL is a significant element of dietary diversity and nutrient sufficiency in adolescents (31). Simultaneously, adolescents who have higher FNL are less likely to be overweight/obese compared with those having lower FNL. People with poor NL tend to consume more fried foods, sugared beverages, red meat, and processed foods, whereas those with good NL consume more vegetables, olive oil, and nuts (10). And a study has shown that increased FNL is associated with lower sugar intake, higher dairy intake, and better energy balance, which positively affect adolescent weight status (30). Prior research has shown that high INL is associated with increased energy score, and high CNL leads to increased consumption of fruits and vegetables (30). In another study (32), INL such as frequency of reading food labels was not a significant predictor of dietary intake. In the current study, INL and CNL were not associated with underweight or overweight/obesity. We proposed several possible reasons for this phenomenon. On the one hand, adolescents may not have enough opportunities to practice knowledge of INL and CNL, as their food habits are determined to a large extent by their schools and parents (33). On the other hand, having high INL/CNL does not mean that the students have the corresponding attitude and can apply knowledge well to critically evaluate nutrition information and handle nutrition problems (34). In this context, schools and teachers should play a leading role in addressing and preventing adolescents' overweight and obesity by providing nutrition education intervention. However, changing intention and behavior is more challenging than changing knowledge (35). Therefore, we should also attend to the influence of the community and families.

In the subgroup analyses, we found that the association between NL and overweight/obesity was consistent. It was suggested that the results were less likely to be confounded by these factors and intervention may work in all subgroups. The only significant interaction we found was between grade and NL with a strong association between NL and overweight/obesity only in senior high school. However, we observed that the NL of junior high school students was higher than that of senior high school students. We did not find a similar study yet, but several explanations were considered for these findings. First, senior high school students have to focus more on their academic subjects (36), and they do not have sufficient time to learn nutrition knowledge. Parents and school staff should realize that health–related behaviors of students directly affect their academic achievement (37). Second, senior high school students may have more self–efficacy and flexibility to translate nutrition knowledge into healthy behaviors (38). A study has revealed that dietary knowledge alone is insufficient to change individual dietary choices (26). Essential behavior capabilities, environmental support, collaborative action, and partnership at multiple levels of influence are all needed to achieve behavior change (39). Therefore, the lack of significant association between NL and overweight/obesity in junior high school students may be due to the failure of nutrition interventions to improve NL as an important mediator between knowledge and practice (26). In brief, strategies and measures need to be adopted to facilitate the ability of junior high school students to apply nutrition knowledge and skills to healthy eating habits. Furthermore, diets of junior high school students may be largely determined by their parents (40). A study has shown that school meals can help students learn about dietary knowledge and skills (41). Our research also showed that boarding school students had higher NL than non–boarding students. Therefore, schools and families may set up supportive environments for lower–grade adolescents to make healthier food choices and sustain behavior change to maintain a healthy weight (42).

This study had several policy implications. Obesity has brought a substantial burden to economic, social, and health. And the Chinese government has made many efforts to curb the incidence of obesity, including the implementation of national policies and programs to promote healthy lifestyles and prevent non-communicable diseases (43). However, the prevalence of overweight/obesity is increasing in China. As promoting NL may improve adolescents' weight status for enhancing their ability to make food choices, perceive food labels, implement food safety precautions, apply healthy cooking methods, and adopt appropriate dietary recommendations (44), it would be of great significance for policymakers, researchers, and other stakeholders in society to assess and develop the NL of adolescents. Meanwhile, our evidence stresses the encouragement to apply nutrition–related knowledge to practical use.

This study had certain limitations. First, this study used cross–sectional survey data and did not permit a reliable inference of causality. Longitudinal studies are necessary to examine the association between NL and overweight/obesity. Second, although quality control was strictly implemented in the process, the online and self–reported survey inevitably brought some information bias. Height and weight were self–reported by the students, which may also introduce biases caused by dishonesty and measurement flaws. Due to the use of online survey, we did not collect information on obesity–related diseases, and thus, students with obesity–related diseases may be included in the study. Third, although we adjusted for gender, grade, ethnicity, residence, primary caregiver, parent's education, and whether boarding in school in the multivariable analysis, residual confounding was still possible. It has been shown that obesity of parents probably may affect the risk of obesity in their offspring due to shared genetic or environmental factors within the family (45). The effect of parental BMI should also be considered in future studies.

In conclusion, this study with a large population–based sample was a representative examination of the association between NL and overweight/obesity among adolescents in Chongqing, applying a specifically developed instrument for the target group. We found that NL was inversely associated with overweight/obesity among adolescents, especially those attending senior high schools. Our results demonstrated that interventions on adolescent obesity may improve their NL and skills. Future study should assess mechanisms such as the effect on eating/physical activity.

## Data Availability Statement

The raw data supporting the conclusions of this article will be made available by the authors, without undue reservation.

## Ethics Statement

The studies involving human participants were reviewed and approved by the Ethics Committee of ChongQing Medical University (approval number: 2021041). Written informed consent to participate in this study was provided by the participants' legal guardian/next of kin.

## Author Contributions

SL and YZ contributed to conception and design of this study. MZ organized the database. SL and ZS performed the statistical analysis. SL, YZ, and ZL wrote the first draft of the manuscript. HZ and ZS wrote sections of the manuscript. All authors contributed to manuscript revision, read, and approved the submitted version.

## Funding

This study was supported by the Chinese Nutrition Society (Science Popularization and Communication Research Fund project, award Number CNS–SCP2020–34).

## Conflict of Interest

The authors declare that the research was conducted in the absence of any commercial or financial relationships that could be construed as a potential conflict of interest.

## Publisher's Note

All claims expressed in this article are solely those of the authors and do not necessarily represent those of their affiliated organizations, or those of the publisher, the editors and the reviewers. Any product that may be evaluated in this article, or claim that may be made by its manufacturer, is not guaranteed or endorsed by the publisher.

## References

[1] Organization WH. Obesity and Overweight. (2021). Available online at: https://www.who.int/news-room/fact-sheets/detail/obesity-and-overweight (accesed June 9, 2021).

[2] NCD-RisC. Worldwide trends in body-mass index, underweight, overweight, and obesity from 1975 to 2016: a pooled analysis of 2416 population-based measurement studies in 128ů9 million children, adolescents, and adults. Lancet. (2017) 390:2627–42. 10.1016/S0140-6736(17)32129-3 29029897 PMC5735219

[3] China NHCotPsRo,. Report on Nutrition Chronic Disease Status of Chinese Residents. (2020) Available online at: http://www.nhc.gov.cn/xcs/s3574/202012/bc4379ddf4324e7f86f05d31cc1c4982.shtml (accessed December 12, 2020)

[4] Simmonds M Llewellyn A Owen CG Woolacott N. Predicting adult obesity from childhood obesity: a systematic review and meta-analysis. Obes Rev. (2016) 17:95–107. 10.1111/obr.12334 26696565

[5] Lobstein T Baur L Uauy R. Obesity in children and young people: a crisis in public health. Obes Rev. (2004) 5 (Suppl. 1):4–104. 10.1111/j.1467-789X.2004.00133.x 15096099

[6] Lee DH Keum N Hu FB Orav EJ Rimm EB Willett WC. Comparison of the association of predicted Fat mass, body mass index, and other obesity indicators with type 2 diabetes risk: two large prospective studies in Us men and women. Eur J Epidemiol. (2018) 33:1113–23. 10.1007/s10654-018-0433-5 30117031

[7] Lee DH Keum N Hu FB Orav EJ Rimm EB Willett WC. Predicted lean body mass, Fat mass, and all cause and cause specific mortality in men: prospective Us cohort study. BMJ. (2018) 362:k2575. 10.1136/bmj.k2575 29970408 PMC6028901

[8] Kalkan I. The impact of nutrition literacy on the food habits among young adults in Turkey. Nutr Res Pract. (2019) 13:352–7. 10.4162/nrp.2019.13.4.352 31388412 PMC6669071

[9] Koca B Arkan G. The relationship between adolescents' nutrition literacy and food habits, and affecting factors. Public Health Nutr. (2020) 29:1–12. 10.1017/S1368980020001494 32723409 PMC11574834

[10] Taylor MK Sullivan DK Ellerbeck EF Gajewski BJ Gibbs HD. Nutrition literacy predicts adherence to healthy/unhealthy diet patterns in adults with a nutrition-related chronic condition. Public Health Nutr. (2019) 22:2157–69. 10.1017/S1368980019001289 31146797 PMC6827561

[11] Taleb S Itani L. Nutrition literacy among adolescents and its association with eating habits and Bmi in Tripoli, Lebanon. Diseases. (2021) 9:25. 10.3390/diseases9020025 33805571 PMC8103266

[12] Krause C Sommerhalder K Beer-Borst S Abel T. Just a subtle difference? findings from a systematic review on definitions of nutrition literacy and food literacy. Health Promot Int. (2018) 33:378–89. 10.1093/heapro/daw084 27803197 PMC6005107

[13] Smith SK Nutbeam D McCaffery KJ. Insights into the concept and measurement of health literacy from a study of shared decision-making in a low literacy population. J Health Psychol. (2013) 18:1011–22. 10.1177/1359105312468192 23676466

[14] Chrissini MK Panagiotakos DB. Health literacy as a determinant of childhood and adult obesity: a systematic review. Int J Adolesc Med Health. (2021) 33:9–39. 10.1515/ijamh-2020-0275 33592684

[15] Shih SF Liu CH Liao LL Osborne RH. Health literacy and the determinants of obesity: a population-based survey of sixth grade school children in Taiwan. BMC Public Health. (2016) 16:280. 10.1186/s12889-016-2879-2 27000035 PMC4802836

[16] Hayba N Khalil C Allman-Farinelli M. Enabling better nutrition and physical activity for adolescents from middle eastern backgrounds: focus groups. Nutrients. (2021) 13:3007. 10.3390/nu13093007 34578885 PMC8466108

[17] Yuen EYN Thomson M Gardiner H. Measuring nutrition and food literacy in adults: a systematic review and appraisal of existing measurement tools. Health Lit Res Pract. (2018) 2:e134–e60. 10.3928/24748307-20180625-01 31294289 PMC6607839

[18] Natour N Al-Tell M Ikhdour O. Nutrition literacy is associated with income and place of residence but not with diet behavior and food security in the Palestinian society. BMC Nutr. (2021) 7:78. 10.1186/s40795-021-00479-3 34789324 PMC8600769

[19] Rosenbaum DL Clark MH Convertino AD Call CC Forman EM Butryn ML. Examination of nutrition literacy and quality of self-monitoring in behavioral weight loss. Ann Behav Med. (2018) 52:809–16. 10.1093/abm/kax052 30124757 PMC6103649

[20] China MoEotPsRo,. Notice on the Issuance of Measures for the Administration of Physical Examination for Primary Secondary School Students. (2021). Available online at: http://www.moe.gov.cn/jyb_xxgk/moe_1777/moe_1779/202110/t20211027_575478.html (accessed September 30, 2021).

[21] Cole TJ Bellizzi MC Flegal KM Dietz WH. Establishing a standard definition for child overweight and obesity worldwide: international survey. BMJ. (2000) 320:1240–3. 10.1136/bmj.320.7244.1240 10797032 PMC27365

[22] Zeng M Wang T Xie C Zhu Y Shi Z Sharma M. Development and Validation of Nutrition Literacy Scale forMiddle School Students in Chongqing, China: A Cross-Sectional Study. (2021). Available online at: https://www.researchgate.net/publication/352754095Development_and_Validation_of_Nutrition_Literacy_Scale_for_Middle_School_Students_in_Chongqing_China_A_Cross-Sectional_Study (accessed June 25, 2021).

[23] Zeng M Xie C Liu Y Cai Z Zhao Y. Evaluation index of nutrition literacy for middle school students in Chongqing based on dephi and analytic hierarchy process method. Health Med Res and Pract. (2021) 18:7–14. 10.11986/j.issn.1673-873X.2021.03.002

[24] Zeng M Zhu Y Cai Z Xian J Li S Wang T. Nutrition literacy of middle school students and its influencing factors: a cross-sectional study in Chongqing, China. Front PubHealth. (2022) 10:807526 10.3389/fpubh.2022.807526 35372191 PMC8965039

[25] Lai IJ Chang LC Lee CK Liao LL. Nutrition literacy mediates the relationships between multi-level factors and college students' healthy eating behavior: evidence from a cross-sectional study. Nutrients. (2021) 13:3451. 10.3390/nu13103451 34684452 PMC8539523

[26] Vaitkeviciute R Ball LE Harris N. The relationship between food literacy and dietary intake in adolescents: a systematic review. Public Health Nutr. (2015) 18:649–58. 10.1017/S1368980014000962 24844778 PMC10271388

[27] Cassar AM Denyer GS O'Connor HT Gifford JA. A qualitative investigation to underpin the development of an electronic tool to assess nutrition literacy in Australians adults. Nutrients. (2018) 10:251. 10.3390/nu10020251 29473889 PMC5852827

[28] Gu X Tucker KL. Dietary quality of the Us child and adolescent population: trends from 1999 to 2012 and associations with the use of federal nutrition assistance programs. Am J Clin Nutr. (2017) 105:194–202. 10.3945/ajcn.116.135095 27881390

[29] Ruiz LD Radtke MD Scherr RE. Development and pilot testing of a food literacy curriculum for high school-aged adolescents. Nutrients. (2021) 13:1532. 10.3390/nu13051532 34062865 PMC8147294

[30] Joulaei H Keshani P Kaveh M. Nutrition literacy as a determinant for diet quality amongst young adolescents: a cross sectional study. Prog Nutr. (2018) 20:455–64. 10.23751/pn.v20i3.6705

[31] Doustmohammadian A Omidvar N Keshavarz-Mohammadi N Eini-Zinab H Amini M Abdollahi M. Low food and nutrition literacy (Fnlit): a barrier to dietary diversity and nutrient adequacy in school age children. BMC Res Notes. (2020) 13:286. 10.1186/s13104-020-05123-0 32532341 PMC7291429

[32] Huang TT Kaur H McCarter KS Nazir N Choi WS Ahluwalia JS. Reading nutrition labels and fat consumption in adolescents. J Adolesc Health. (2004) 35:399–401. 10.1016/S1054-139X(04)00070-9 15488434

[33] Akman M Tuzun S Unalan P. Healthy Eating Patterns and Physical Activity Status of Adolescents [2021-01]. Available online at: https://www.researchgate.net/publication/289632833_Healthy_eating_patterns_and_physical_activity_status_of_adolescents.

[34] Ronto R Ball L Pendergast D Harris N. Food literacy at secondary schools in Australia. J Sch Health. (2016) 86:823–31. 10.1111/josh.12440 27714873

[35] Hawkins M Watts E Belson SI Snelling A. Design and implementation of a 5-year school-based nutrition education intervention. J Nutr Educ Behav. (2020) 52:421–8. 10.1016/j.jneb.2019.12.005 31948742

[36] Ronto R Ball L Pendergast D Harris N. What is the status of food literacy in Australian high schools? perceptions of home economics teachers. Appetite. (2017) 108:326–34. 10.1016/j.appet.2016.10.024 27771492

[37] Rasberry CN Tiu GF Kann L McManus T Michael SL Merlo CL. Health-Related behaviors and academic achievement among high school students - United States, 2015. MMWR Morb Mortal Wkly Rep. (2017) 66:921–7. 10.15585/mmwr.mm6635a1 28880853 PMC5689037

[38] Ciarrochi J Sahdra B Marshall S Parker P Horwath C. Psychological flexibility is not a single dimension: The distinctive flexibility profiles of underweight, overweight, and obese people. J Context Behav Sci. (2014) 3:236–47. 10.1016/j.jcbs.2014.07.002

[39] Hawkes C Smith TG Jewell J Wardle J Hammond RA Friel S. Smart food policies for obesity prevention. Lancet. (2015) 385:2410–21. 10.1016/S0140-6736(14)61745-1 25703109

[40] Gibbs HD Kennett AR Kerling EH Yu Q Gajewski B Ptomey LT. Assessing the nutrition literacy of parents and its relationship with child diet quality. J Nutr Educ Behav. (2016) 48:505–9.e1. 10.1016/j.jneb.2016.04.006 27216751 PMC4931947

[41] Benn J Carlsson M. Learning through school meals? Appetite. (2014) 78:23–31. 10.1016/j.appet.2014.03.008 24656948

[42] Swinburn B Kraak V Rutter H Vandevijvere S Lobstein T Sacks G. Strengthening of accountability systems to create healthy food environments and reduce global obesity. Lancet. (2015) 385:2534–45. 10.1016/S0140-6736(14)61747-5 25703108

[43] China SCotPsRo,. National Nutrition Plan 2017–2030. (2017). Available online at: https://extranet.who.int/nutrition/gina/en/node/24710 (accessed September 30, 2021).

[44] Chung L. Food literacy of adolescents as a predictor of their healthy eating and dietary quality. J Child Adolesc Behav. (2017) 5:3. 10.4172/2375-4494.1000e117

[45] Whitaker RC Wright JA Pepe MS Seidel KD Dietz WH. Predicting obesity in young adulthood from childhood and parental obesity. N Engl J Med. (1997) 337:869–73. 10.1056/NEJM19970925337130 9302300

