# Supplementary material for: Association Between Nutrition Literacy and Overweight/Obesity of Adolescents: A Cross–Sectional Study in Chongqing, China
Source: Front Nutr. 2022 May 12;9:893267. doi: 10.3389/fnut.2022.893267 (PMC9134066; doi:10.3389/fnut.2022.893267)
Supplement: Supplementary file 1 [file Table_1.DOCX]

**Section B of ‘CM-NLS’**

|  | **Scale items** /**Question** | **option** |
| --- | --- | --- |
|  | **1.** **Functional NL** |  |
|  | *1.1 obtain* |  |
| Q1_1.1.1 | I will seek answers when I don't know anything about nutrition. **[liket-5]** | ①Strongly disagree ②Disagree ③Neutral ④Agree ⑤Strongly agree |
| Q2_1.1.2 | Know where to find accurate information when I have a nutrition-related problem or want to learn healthy eating behaviors. **[liket-5]** | ①Strongly disagree ②Disagree ③Neutral ④Agree ⑤Strongly agree |
| Q3_1.1.3 | It is not difficult for me to find the need nutritional information from a large number of information sources. **[liket-5]** | ①Strongly disagree ②Disagree ③Neutral ④Agree ⑤Strongly agree |
|  | *1.2 understand* |  |
| Q4_1.2.1 | Learn about food sources and categories **[sorting problem]** | 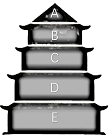①Pork, chicken, duck, fish, eggs, etc.  ②Rice noodles, sweet potatoes, mung beans, etc. ③Milk and milk products, soybeans and their products ④Edible oil, salt ⑤Vegetables and fruits |
| Q5_1.2.2 | Whole grains (millet, corn, etc.) are more nutritious than refined grains (rice, flour, etc.). **T** | 1. True ②False ③Don’t know |
| Q6_1.2.3 | Know the advantage that eats bean curd, soya-bean milk to wait for soybean product**[multi-select]** | ①Good for health ②Good for patients with cardiovascular disease ③Increase the intake of high-quality protein ④Prevent the adverse effects of excessive consumption of meat ⑤Don’t know |
| Q7_1.2.4 | Smoking and salting foods can increase the risk of cancer. **T** | 1. True ②False ③Don’t know |
| Q8_1.2.5 | Being overweight or underweight increases the risk of disease. **T** | 1. True ②False ③Don’t know |
| Q9_1.2.6 | When eating meat, try to eat lean meat, fat meat can eat, but cannot eat more. **T** | 1. True ②False ③Don’t know |
| Q10_1.2.7 | Good eating habits can prevent chronic diseases such as hypertension and diabetes. **T** | 1. True ②False ③Don’t know |
| Q11_1.2.8 | You can reduce your exercise if you eat less. **F** | 1. True ②False ③Don’t know |
| Q12_1.2.9 | Drink water in small quantities more than once. **T** | 1. True ②False ③Don’t know |
| Q13_1.2.10 | Separate meals can help prevent mouth to mouth diseases. **T** | 1. True ②False ③Don’t know |
| Q14_1.2.11 | It is easy to understand the contents of the Dietary Guidelines for Chinese residents. **[liket-5]** | ①Never heard ②Strongly disagree ③Disagree ④Neutral ⑤Agree ⑥Strongly agree |
| Q15_1.2.12 | It is easy to understand the nutritional information (such as energy, protein, sugar, etc.) on food packaging. **[liket-5]** | 1. Strongly disagree ②Disagree ③Neutral ④Agree ⑤Strongly agree |
| Q16_1.2.13 | It is easy to understand that recommendations relating to health and nutrition in secondary school students. **[liket-5]** | 1. Strongly disagree ②Disagree ③Neutral ④Agree ⑤Strongly agree |
| Q17_1.2.14 | It is easy to understand nutrition information you read in a brochure, book or on the Internet. **[liket-5]** | 1. Strongly disagree ②Disagree ③Neutral ④Agree ⑤Strongly agree |
|  | *1.3 apply/use* |  |
| Q18_1.3.1 | When eating, consider **nutrition** first. **[single-select]** | 1. Taste ②Nutrition ③Satisfy hunger ④Maintain body ⑤Other ⑥No standard |
| Q19_1.3.2 | Use **nutrition labels** to choose foods wisely. **[multi-select]** | ①Production date ②expiration date ③Nutrient content table ④Manufacturer ⑤Don’t know ⑥I haven’t paid attention to it |
| Q20_1.3.3 | Choose snacks wisely. **[multi-select]** | This option can be modified according to the food culture habits of each country. |
| Q21_1.3.4 | Don't substitute fruits for vegetables. **[liket-5]** | 1. Never experienced ②Never ③Occasionally ④Sometimes ⑤Always |
| Q22_1.3.5 | Don't replace fresh vegetables with pickles and pickles. **[liket-5]** | 1. Never experienced ②Never ③Occasionally ④Sometimes ⑤Always |
| Q23_1.3.6 | Don't snack instead of meals. **[liket-5]** | 1. Never experienced ②Never ③Occasionally ④Sometimes ⑤Always |
| Q24_1.3.7 | Do not use canned fruit, preserved fruit and other processed fruit products instead of fresh fruit. **[liket-5]** | 1. Never experienced ②Never ③Occasionally ④Sometimes ⑤Always |
| Q25_1.3.8 | Cooked food should be kept at room temperature for no more than 2 hours. **[liket-5]** | 1. Never experienced ②Never ③Occasionally ④Sometimes ⑤Always |
| Q26_1.3.9 | Eat a variety of foods: eat at least 12 foods a day and 25 foods a week. **[single-select]** | Give an example, ask the respondent to fill in the type of food consumed in the past 24 hours ①0~4 ②5~8 ③9~11 ④≥12 ⑤Don’t know |
| Q27_1.3.10 | Eat breakfast every day. **[single-select]** | The frequency of eating breakfast in the past week①0/weekly ②1~2/weekly ③3~4/weekly ④5~6/weekly ⑤Every day |
| Q28_1.3.11 | Eat fruit every day. **[single-select]** | The frequency of eating fresh fruit in the past week①0/weekly ②1/weekly ③2~6 /weekly ④1 /day ⑤≥2 /day |
| Q29_1.3.12 | Drink milk every day. **[single-select]** | The frequency of drinking milk in the past week①0/weekly ②1/weekly ③2~6 /weekly ④1 /day ⑤≥2 /day |
| Q30_1.3.13 | Avoid or limit sugary drinks. **[single-select]** | The frequency of drinking sugary drinks in the past week①0/weekly ②1/weekly ③2~6 /weekly ④1 /day ⑤≥2 /day |
| Q31_1.3.14 | Don't drink. **[single-select]** | 1. Yes ②No |
| Q32_1.3.15 | Cut down on Western fast food. **[single-select]** | The frequency of eating Western fast food in the past week①0/weekly ②1/weekly ③2~6 /weekly ④1 /day ⑤≥2 /day |
| Q33_1.3.16 | Cut down on fat, smoke and spicy foods. **[single-select]** | The frequency of eating fat, smoke and spicy foods in the past week①0/weekly ②1/weekly ③2~6 /weekly ④1 /day ⑤≥2 /day |
| Q34_1.3.17 | Do not patronize food and drink stalls such as roadside stands. **[liket-5]** | 1. Never experienced ②Never ③Occasionally ④Sometimes ⑤Always |
| Q35_1.3.18 | Knowledge of healthy weight and can rightly realize body shape. **[single-select]** | Respondents were asked to evaluate their body shape after self-reporting their height(m) and weight(kg) ①Thin ②Normal ③Overweight ④Obese ⑤Don’t know |
|  | **2. Interactive NL** |  |
|  | *2.1 interact* |  |
| Q36_2.1.1 | An active attitude toward obtaining nutritional information. **[liket-5]** | 1. Never experienced ②Never ③Occasionally ④Sometimes ⑤Always |
| Q37_2.1.2 | The willingness communicate to learn about nutrition and health. **[liket-5]** | 1. Strongly disagree ②Disagree ③Neutral ④Agree ⑤Strongly agree |
| Q38_2.1.3 | The willingness to receive nutrition education. **[liket-5]** | 1. Strongly disagree ②Disagree ③Neutral ④Agree ⑤Strongly agree |
| Q39_2.1.4 | The willingness to change poor eating habits using the nutrition knowledge learned. **[liket-5]** | 1. Strongly disagree ②Disagree ③Neutral ④Agree ⑤Strongly agree |
| Q40_2.1.5 | The willingness to persuade others to change their bad eating habits. **[liket-5]** | 1. Strongly disagree ②Disagree ③Neutral ④Agree ⑤Strongly agree |
|  | **3.** **Critical NL** |  |
|  | *3.1 medial literacy(ML)* |  |
| Q41_3.1.1 | Talk about nutrition with others (e.g. friends, family, etc.). **[liket-5]** | 1. Strongly disagree ②Disagree ③Neutral ④Agree ⑤Strongly agree |
| Q42_3.1.2 | Take the initiative to disseminate nutrition knowledge to others. **[liket-5]** | ①Never experienced ②Never ③seldom ④Occasionally ⑤Sometimes ⑥Always |
| Q43_3.1.3 | Attention to nutritional information in the media. **[liket-5]** | ①Never experienced ②Never ③seldom ④Occasionally ⑤Sometimes ⑥Always |
| Q44_3.1.4 | Critical of nutrition information from all sources in society. **[liket-5]** | 1. Never experienced ②Never ③seldom ④Occasionally ⑤Sometimes ⑥Always |
| Q45_3.1.5 | I can judge the accuracy and scientific nature of nutrition-related information in the media. **[liket-5]** | 1. Never experienced ②Never ③seldom ④Occasionally ⑤Sometimes ⑥Always |
| Q46_3.1.6 | When confronted with a great amount of nutritional information, I can judge whether the information is right or wrong through media reports. **[liket-5]** | 1. Never experienced ②Never ③seldom ④Occasionally ⑤Sometimes ⑥Always |
| Q47_3.1.7 | The extent to which nutritional information in the media affects you. **[liket-5]** | 1. Never experienced ②Never ③seldom ④Occasionally ⑤Sometimes ⑥Always |
| Q48_3.1.8 | Dare to question deeply rooted social and cultural phenomena related to food and health. **[liket-5]** | ① Never experienced ②Never ③seldom ④Occasionally ⑤Sometimes ⑥Always |
|  | *3.2 critical* |  |
| Q49_3.2.1 | How easy it is to distinguish whether nutritional information is scientific or not. **[liket-5]** | ①Never experienced ②Very difficult ③Slightly difficult ④General ⑤Slightly simple ⑥Very simple |
| Q50_3.2.2 | How easy it is to distinguish between healthy and less healthy foods. **[liket-5]** | ①Never experienced ②Very difficult ③Slightly difficult ④General ⑤Slightly simple ⑥Very simple |
| Q51_3.2.3 | How easy it is to assess the impact of eating habits on health. **[liket-5]** | ①Never experienced ②Very difficult ③Slightly difficult ④General ⑤Slightly simple ⑥Very simple |
| Q52_3.2.4 | When I am given advice on healthy eating, I can judge what fits my health needs. **[liket-5]** | ①Never experienced ②Very difficult ③Slightly difficult ④General ⑤Slightly simple ⑥Very simple |
